# Predictors of response to low-dose amitriptyline for irritable bowel syndrome and efficacy and tolerability according to subtype: *Post hoc* analyses from the ATLANTIS trial

**DOI:** 10.1136/gutjnl-2024-334490

**Published:** 2025-01-25

**Authors:** Alexandra Wright-Hughes, Pei-Loo Ow, Sarah L. Alderson, Matthew J. Ridd, Robbie Foy, Felicity L. Bisho, Matthew Chaddock, Catherine Fernandez, Elspeth A. Guthrie, Delia P. Muir, Christopher A. Taylor, Amanda J. Farrin, Hazel A. Everitt, Alexander C. Ford, ATLANTIS trialists

**Affiliations:** 1 Clinical Trial Research Unit, Leeds Institute of Clinical Trials Research, School of Medicine, University of Leeds, UK; 2 Leeds Institute of Health Sciences, School of Medicine, University of Leeds, UK; 3 Population Health Sciences, Bristol Medical School, University of Bristol, UK; 4 Centre for Clinical and Community Applications of Health Psychology, School of Psychology, University of Southampton, UK; 5 Let’sCure IBS, Leeds, UK; 6 Primary Care Research Centre, Faculty of Medicine, University of Southampton, UK; 7 Leeds Institute of Medical Research at St. James’s, University of Leeds, Leeds, UK; 8 Leeds Gastroenterology Institute, St James’s University Hospital, Leeds, UK

**Keywords:** irritable, bowel, syndrome, tricyclic, antidepressant, abdominal, pain, bowel, habit, abdominal, distension

## Abstract

**Background:**

Low-dose amitriptyline, a tricyclic antidepressant (TCA), was superior to placebo for irritable bowel syndrome (IBS) in the ATLANTIS trial.

**Objective:**

To perform *post hoc* analyses of ATLANTIS for predictors of response to, and tolerability of, a TCA.

**Design:**

ATLANTIS randomized 463 adults with IBS to amitriptyline (232) or placebo (231). We examined effect of baseline demographic and disease-related patient characteristics on response to amitriptyline, and effect of amitriptyline on individual symptoms and side effects by subtype.

**Results:**

There was a quantitative difference in amitriptyline effectiveness in those ≥50 years versus <50 on the IBS severity scoring system (IBS-SSS) (interaction *p*=0.048, mean difference in ≥50 years subgroup -46.5; 95% CI -74.2 to -18.8, *p*=0.0010), and subjective global assessment of relief (interaction *p*=0.068, odds ratio (OR) in ≥50 years subgroup 2.59; 95% CI 1.47 to 4.55, *p*=0.0010), and those in the 70% most deprived areas of England compared with the 30% least deprived for a ≥30% improvement in abdominal pain (interaction *p*=0.021, OR in 70% most deprived subgroup 2.70; 95% CI 1.52 to 4.77, *p*=0.0007). Stronger treatment effects were seen in men, with higher Patient Health Questionnaire-12 scores, and with IBS with diarrhoea. Mean differences in individual IBS-SSS components favoured amitriptyline, and side effects were similar, across almost all IBS subtypes.

**Conclusion:**

These exploratory analyses demonstrate there are unlikely to be deleterious effects of amitriptyline in any particular IBS subtype and could help identify patients in whom amitriptyline may be more effective. Clinical trial registration: ISRCTN48075063.

## Abbreviations

ASEC: Antidepressant Side Effect Checklist
CI: confidence interval
EMA: European Medicines Agency
FDA: Food and Drug Administration
GP: general practitioner
HADS: Hospital Anxiety and Depression Scale
IBS: irritable bowel syndrome
IBS-C: irritable bowel syndrome with constipation
IBS-D: irritable bowel syndrome with diarrhoea
IBS-M: irritable bowel syndrome with mixed bowel habits
IBS-U: irritable bowel syndrome unclassified
IBS-SSS: Irritable Bowel Syndrome Severity Scoring System
IMD: index of mean deprivation
MD: mean difference
OR: odds ratio
PHQ-12: Patient Health Questionnaire-12
RCT: randomised controlled trial
SGA: subjective global assessment
TCA: tricyclic antidepressant
WSAS: Work and Social Adjustment Scale

## Introduction

Irritable bowel syndrome (IBS) is a disorder of gut-brain interaction,[1] characterised by abdominal pain with disordered stool form or stool frequency.[2] IBS affects 5% of the population globally.[3, 4] Patients are subtyped according to predominant bowel habit into constipation (IBS-C), diarrhoea (IBS-D), or mixed bowel habits (IBS-M), or meeting criteria for none of these three, so-called IBS-unclassified (IBS-U). As IBS is chronic and relapsing,[5] it represents a substantial burden to both individuals and society. IBS has a negative impact on quality of life,[6] as well as ability to work and socialise.[7] Patients are willing to accept considerable risk in return for possible cure of symptoms.[8] In the UK, estimated direct costs attributable to IBS are around £1.3 billion per year.[9]

First-line treatments include dietary changes, antispasmodics, laxatives, or anti-diarrheal drugs.[10] However, many patients do not experience adequate relief of symptoms with these. Prior evidence from meta-analyses of randomised controlled trials (RCTs) suggests that tricyclic antidepressants (TCAs) may be beneficial in IBS,[11–13] perhaps due to effects on gastrointestinal motility and pain sensation.[14–16] Whether some beneficial effects are affected by other factors is unclear though, because most trials have been small and unable to examine these issues. In addition, because of their impact on gastrointestinal motility, TCAs may have deleterious effects on symptoms for some patients, such as exacerbating constipation in those with IBS-C or IBS-M.

Recently, we reported the results of ATLANTIS, a pragmatic, definitive, RCT of the TCA amitriptyline at low-dose for IBS.[17] At 6-month follow-up, amitriptyline was more effective than placebo across a range of symptom measures. Because ATLANTIS recruited over 450 patients, *post hoc* analysis is not only feasible but may also yield valuable insights into potential predictors of response to TCAs, the effect of active drug on the individual symptoms of IBS, and tolerability according to IBS subtype. We, therefore, performed exploratory analyses to assess effectiveness of low-dose amitriptyline versus placebo according to patient characteristics and to investigate consistency of treatment effects across subgroups of clinical importance,[18] and on individual symptoms of IBS by subtype. We also assessed the safety profile of amitriptyline by IBS subtype. We hypothesised there would be consistent predictors of response and that, because of the low doses used in ATLANTIS, amitriptyline would have a beneficial effect on individual gastrointestinal symptoms of IBS, and would be well-tolerated, irrespective of subtype.

## Methods

### Study Design

This is a secondary analysis of data from ATLANTIS (AmitripTyline at Low-dose ANd Titrated for Irritable bowel syndrome as Second-line treatment). ATLANTIS was an individually randomised, double-blind, superiority trial examining the effectiveness of 6 months of low-dose (10mg-30mg/day) amitriptyline versus placebo as second-line treatment for adults with IBS in primary care. The trial was conducted in 55 general practices in three geographical regions, or hubs, in England: West Yorkshire; Wessex; and the West of England. The study protocol and all subsequent amendments were approved by Yorkshire and the Humber (Sheffield) Research Ethics Committee (19/YH/0150) and fully published.[19] The methodology and main results are reported elsewhere.[17, 19–21] Details concerning participants, randomisation, masking, procedures, and outcome measures are, therefore, provided in the Supplement. ATLANTIS was conducted in accordance with the principles of Good Clinical Practice and the Declaration of Helsinki and registered with the ISRCTN (ISRCTN48075063).

### Baseline Data

A total of 10 baseline measurements defined the candidate effect modifiers of interest. Categorical variables included sex and IBS subtype (IBS-C vs. IBS-D vs. IBS-M or IBS-U). Continuous measurements included age, deprivation of regional residence as assessed by index of mean deprivation (IMD) decile at the participant level, IBS severity at baseline on the IBS severity scoring system (IBS-SSS),[22] time from diagnosis, IBS-associated somatic symptoms,[23] assessed using the Patient Health Questionnaire-12 (PHQ-12),[24] anxiety and depression scores via the Hospital Anxiety and Depression Scale (HADS),[25] and ability to work and participate in other activities measured using the Work and Social Adjustment Scale (WSAS).[26] The total PHQ-12 score ranges from a minimum of 0 to a maximum of 24 for women and 0 to 22 for men, with higher scores indicating greater somatic symptoms.[24] The total HADS score ranges from 0 to 21 for both anxiety and depression with scores 0-7 defining normal, 8-10 borderline, and ≥11 abnormal scores.[25] The total WSAS score ranges from 0 to 40, with <10 used to define low, 10 to 20 moderate, and >20 severe impairment.[26] IBS is considered a biopsychosocial disorder,[27] and all the effect modifiers are relevant in this regard as they include age, biological sex, social circumstances (deprivation index), psychological impact, and impact on work and social activities.

### Statistical Analysis

All authors had access to study data and reviewed and approved the final manuscript. All analyses were exploratory and were performed in the intention-to-treat population, defined as all participants randomised to trial medication, regardless of adherence. All statistical testing used two-sided 5% significance levels, performed in SAS, version 9.4.

We compared 6-month total IBS-SSS scores, subjective global assessment (SGA) of relief of IBS symptoms at 6 months, and a ≥30% improvement in abdominal pain at 6 months in each trial arm according to the 10 candidate effect modifiers at trial entry. We further compared individual components of the IBS-SSS score at 6 months, including frequency of abdominal pain, severity of abdominal pain, severity of abdominal distension, satisfaction with bowel habit, and degree to which IBS symptoms were affecting, or interfering with, the participant’s life in general between amitriptyline and placebo according to IBS subtype only.

For each candidate effect modifier, we analysed the outcome using linear (IBS-SSS score) or logistic (SGA of relief or a ≥30% improvement in abdominal pain) regression, as appropriate, with fixed effects for randomisation strata (IBS subtype, HADS depression score <8 or ≥8, recruitment hub), IBS-SSS score at baseline (or IBS-SSS component scores, where appropriate; i.e. analysis of covariance),[28] effect modifier, treatment and effect modifier x treatment interaction. We imputed missing data by treatment arm via multiple imputation by chained equations with 25 imputations, including recruitment hub, IBS subtype, sex, age, baseline questionnaire scores (IBS-SSS, PHQ-12, HADS, and WSAS), 3-month outcome as appropriate, and 6-month treatment status in the model.

Simple effects, together with 95% confidence intervals (CIs) and *p*-values, of the adjusted mean difference (MD) and odds ratio (OR), as appropriate to outcome, summarised the treatment effect (amitriptyline vs. placebo) within each category of the effect modifier, or at distinct levels of continuous effect modifiers.[29] Alongside simple effects illustrating the relative effects within subgroups, the *p*-value for the interaction term was used to assess whether there was evidence of differential treatment effectiveness across patient characteristics (i.e., whether the intervention was more or less effective according to the effect modifier). To explore placebo effects, simple effects were also extracted to summarise effect modifiers within each trial arm on likelihood of SGA of relief of IBS symptoms or a ≥30% improvement in abdominal pain at 6 months. Given the exploratory nature of the analysis, the findings are intended to guide future research and were not adjusted for multiple testing.

To provide clinically clear and communicable results, aligned with applications to precision medicine, we categorised continuous baseline effect modifiers to allow treatment effects to be estimated within subgroups together with the treatment by subgroup interaction. We categorised effect modifiers primarily according to recommended cut offs, where these were available, including for the HADS (normal vs. borderline or abnormal), WSAS (low vs. moderate vs. severe impairment), and IBS-SSS (mild vs. moderate vs. severe symptoms), and elsewhere using the median, including for age (<50 vs. ≥50 years), PHQ-12 score (≤6 vs. >6), aligning with the suggested cut-off to distinguish IBS from non-IBS symptoms,[24] IMD decile (≤7 vs. >7), and time from diagnosis of IBS (≤10 vs. >10 years). We conducted sensitivity analysis of these effect modifiers on the continuous scale, in which the estimated interaction effect represents the change in treatment effect for a one-unit increase in the continuous effect modifier, in order to respect the underlying data structure and protect against loss of information and statistical power due to categorisation.[30]

Descriptive statistics of self-reported treatment-emergent adverse events on the Antidepressant Side Effect Checklist (ASEC), and total ASEC score, at 6 months were presented by IBS subtype according to medication received for participants still on treatment.

## Results

Between 18^th^ October 2019 and 11^th^ April 2022, we randomised 463 participants to amitriptyline (n=232) or placebo (n=231). Trial follow-up completed in October 2022 with 6-month follow-up achieved for 401 (87%) participants (204 (88%) amitriptyline, 197 (85%) placebo), and completion of 6 months of treatment in 338 (73%) participants (173 (75%) amitriptyline and 165 (71%) placebo). As previously reported, amitriptyline was superior to placebo at 6 months in the intention-to-treat analysis for the primary outcome (MD in IBS-SSS - 27.0; 95% CI -46.9 to -7.1, *p*=0.0079), key secondary outcome of SGA of relief (OR 1.78; 95% CI 1.19 to 2.66, *p*=0.0050), and the exploratory outcome of a ≥30% reduction in abdominal pain (OR 1.66; 95% CI 1.12 to 2.46, *p*=0.012).[17, 21]

According to the candidate effect modifiers of interest, the median age of participants was 50 years, with a median IMD decile of 7 (indicating that half the sample were in the 30% least deprived areas in England), a median PHQ-12 score of 6, and a median time since IBS diagnosis of 10 years. Most participants were female (68.0%); with IBS-M or IBS-U (44.3%), followed by IBS-D (39.1%), or IBS-C (16.6%), and reported moderate (43.4%) or severe (41.3%) symptoms on the IBS-SSS. Anxiety and depression scores were normal in 52.9% and 84.2% of all participants, respectively. Impairment in work and other activities was low (47.4%) or moderate (39.3%) for most participants.

### Effect of Baseline Characteristics on Response to Amitriptyline at 6 Months

Based on the *p*-value for the treatment by effect modifier interaction (Table 1), there was a quantitative difference in the effectiveness of amitriptyline according to age on the IBS-SSS (interaction *p*=0.048), with improved treatment effects in participants aged ≥50 years (MD -46.5; 95% CI -74.2 to -18.8, *p*=0.0010). This effect was also observed for SGA of relief at the 10% significance level (interaction *p*=0.068) with an increased odds of response for SGA of relief with amitriptyline compared with placebo in participants aged ≥50 years (OR 2.59; 95% CI 1.47 to 4.55, *p*=0.0010) (Table 1). Although there was a similarly increased odds for a ≥30% improvement in abdominal pain in participants aged ≥50 years (OR 2.10; 95% CI 1.20 to 3.68, *p*=0.0093) this was not significantly different to that in participants aged <50 years (interaction *p*=0.23) (Table 2). There was also evidence of a quantitative difference in the effectiveness of amitriptyline according to IMD for a ≥30% improvement in abdominal pain (interaction *p*=0.021), with an increased odds of response to amitriptyline compared with placebo in participants living in the 70% most deprived areas in England (OR 2.70; 95% CI 1.52 to 4.77, *p*=0.0007). There was a similarly increased treatment effect in participants living in the 70% most deprived areas on the IBS-SSS (MD -39.1; 95% CI -66.6 to -11.6, *p*=0.0054) and SGA of relief (OR 2.29; 95% CI 1.30 to 4.03, *p*=0.0043), but this was not significantly different to hat in participants in the 30% least deprived areas (interaction *p*=0.33 and *p*=0.29, respectively). No qualitative effect modifier effects were observed, in which amitriptyline was suggested to be beneficial in one effect modifier subgroup but harmful in another.

**Figure 1:**
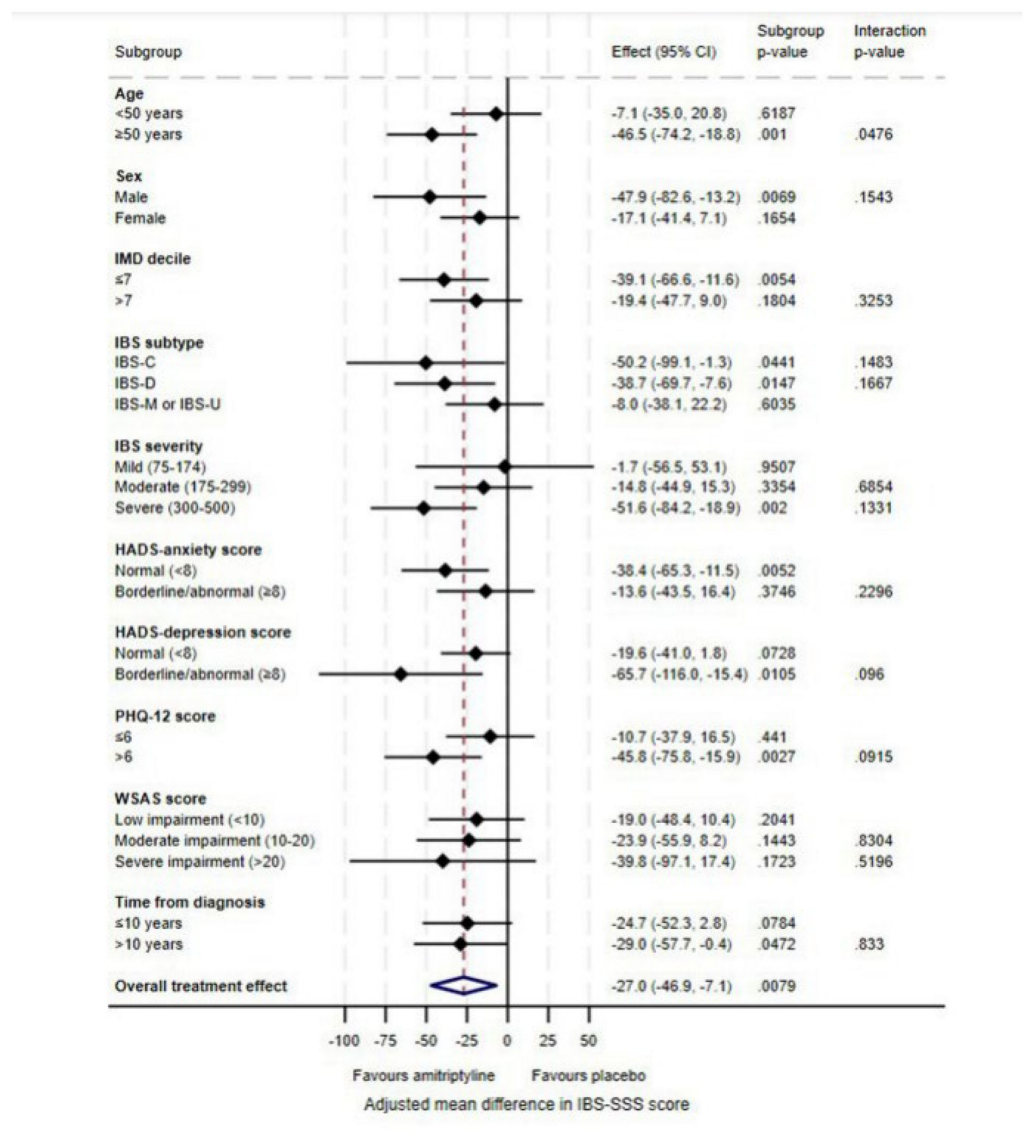
Forest Plot of Treatment Effects on the Total IBS-SSS score at 6 Months According to Participant Baseline Characteristics.

**Figure 2:**
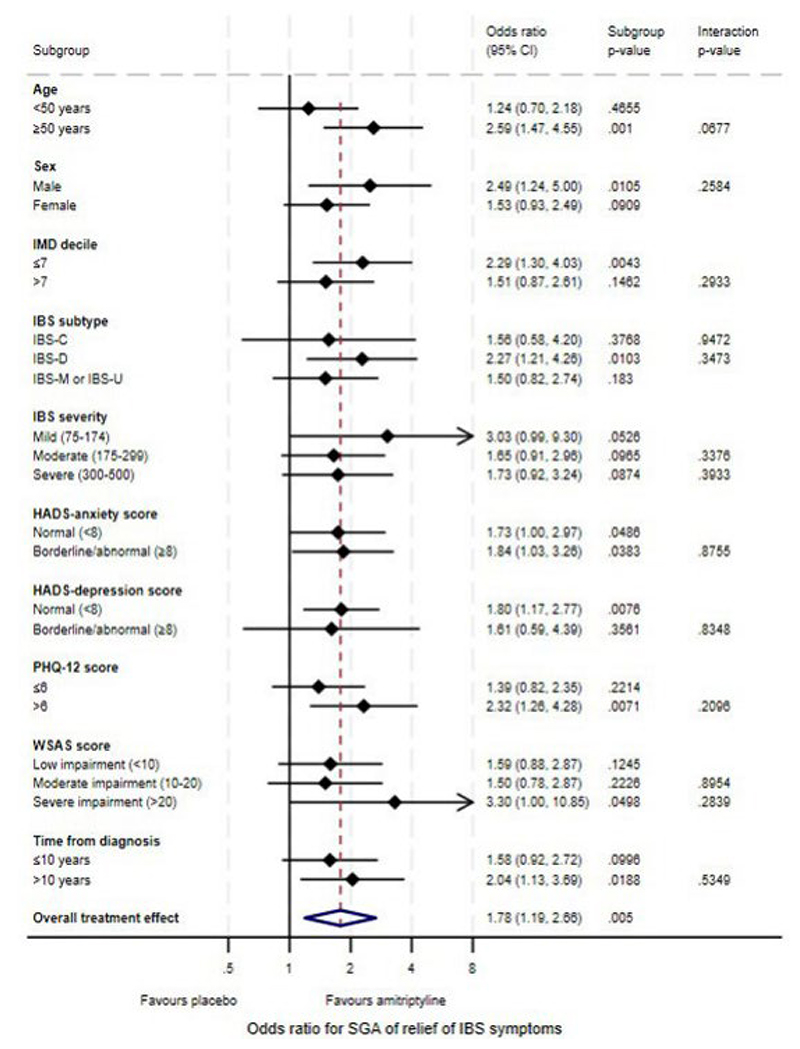
Forest Plot of Treatment Effects on SGA of Relief of IBS Symptoms at 6 Months According to Participant Baseline Characteristics.

**Figure 3:**
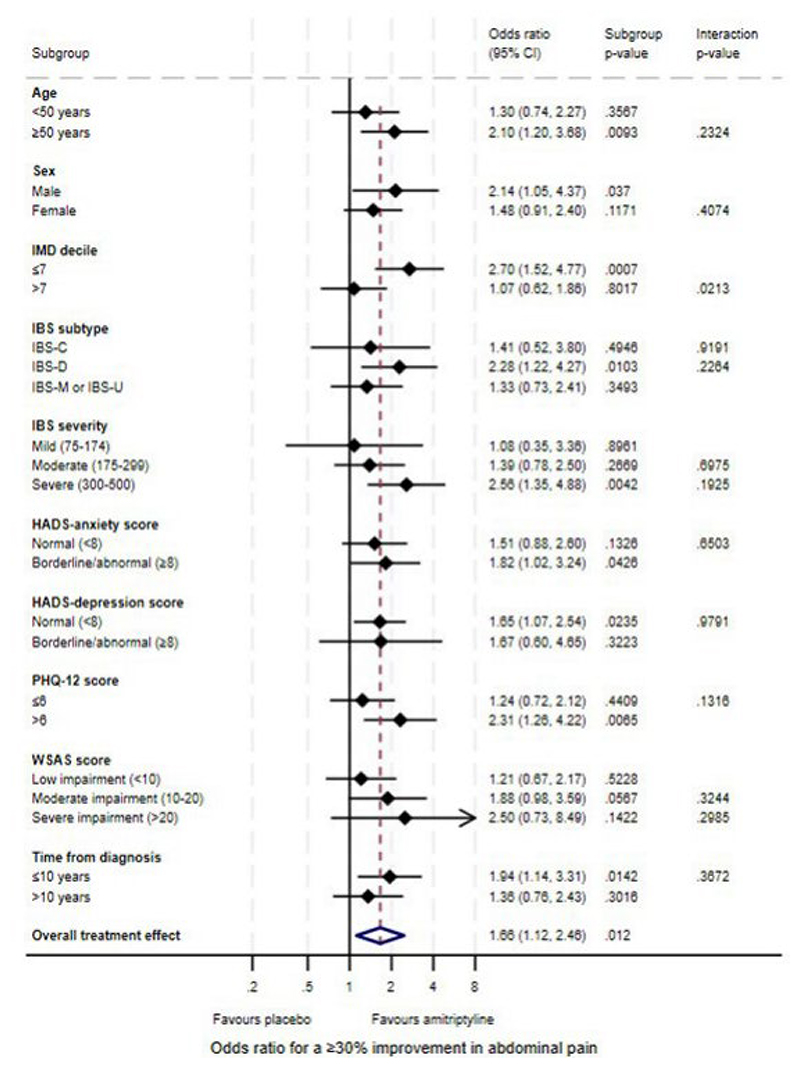
Forest Plot of Treatment Effects on a ≥30% Improvement in Abdominal Pain at 6 Months According to Participant Baseline Characteristics.

There was some evidence of a differential treatment effect on the IBS-SSS for those with above median PHQ-12 scores (MD -45.8; 95% CI -75.8 to -15.9, *p*=0.0027). This was consistent for odds of SGA of relief (OR 2.32; 95% CI 1.26 to 4.28, *p*=0.0071) and a ≥30% improvement in abdominal pain (OR 2.31; 95% CI 1.26 to 4.22, *p*=0.0065). Stronger treatment effects were observed consistently for all three endpoints in men (IBS-SSS MD -47.9; 95% CI -82.6 to -13.2, *p*=0.0069, OR for SGA of relief 2.49; 95% CI 1.24 to 5.00, *p*=0.011, and OR for a ≥30% improvement in abdominal pain 2.14; 95% CI 1.05 to 4.37, *p*=0.037) (Tables 1 and 2, Figures 1, 2, and 3). There were also stronger treatment effects observed in those with severe symptoms on the IBS-SSS (MD -51.6; 95% CI -84.2 to -18.9, *p*=0.0020) and a ≥30% improvement in abdominal pain (OR 2.56; 95% CI 1.35 to 4.88, *p*=0.0042) but not SGA of relief, where those with mild symptoms exhibited the greatest treatment effect (OR 3.03; 95% CI 0.99 to 9.30, *p*=0.053). The strongest treatment effects were also observed in participants with IBS-D for SGA of relief (OR 2.27; 95% CI 1.21 to 4.26, *p*=0.010), and a ≥30% improvement in abdominal pain (OR 2.28; 95% CI 1.22 to 4.27, *p*=0.010), and in participants with IBS-C or IBS-D on the IBS-SSS (IBS-C MD -50.2; 95% CI -99.1 to -1.3, *p*=0.044, IBS-D MD -38.7; 95% CI -69.7 to - 7.6, *p*=0.015).

In the sensitivity analysis evaluating the effectiveness of amitriptyline according to effect modifiers modelled on the continuous scale (Supplementary Figures 1 and 2), no statistically significant effects were observed across any outcome. Where significant moderating effects were detected on dichotomised variables of age, IMD decile, HADS-depression, or PHQ-12 scores, sensitivity analyses on the continuous scale were consistent in terms of a trend towards improved treatment effects in older participants, those living in more deprived areas, and those with higher PHQ-12 scores. However, these interaction effects were not statistically significant. Analyses of the effect of baseline characteristics on response to either amitriptyline or placebo at 6 months are provided in the Supplement.

### Effect of Amitriptyline on Individual Components of the IBS-SSS According to IBS Subtype

Estimated adjusted mean differences in individual IBS-SSS components favoured amitriptyline across all subtypes (Table 3), with the exception of abdominal pain frequency in participants with IBS-M or IBS-U, where there was a small and non-significant MD in favour of placebo (2.5; 95% CI -5.5 to 10.5, *p*=0.54). The treatment effect on abdominal pain was significant (interaction *p*=0.045) in the IBS-D subset (MD -9.2; 95% CI -17.4 to -1.0, *p*=0.028); although it was similarly improved in the IBS-C subset (MD -8.9; 95% CI -22.1 to 4.2, *p*=0.18) this was not significantly different to those with IBS-M or IBS-U (interaction *p*=0.14) due to the smaller sample size. No further significant treatment-subtype interaction effects were observed.

However, smaller treatment effects were observed consistently across all components of the IBS-SSS in those with IBS-M or IBS-U compared with those with IBS-D or IBS-C, as was observed for the total IBS-SSS, SGA of relief, and a ≥30% improvement in abdominal pain outcomes. In addition to findings for abdominal pain frequency, significant differences vs. placebo were also observed for abdominal distension severity in those with IBS-C (-15.0; 95% CI -27.6 to -2.3, *p*=0.021), satisfaction with bowel habit in those with IBS-C (-13.6; 95% CI - 26.0 to -1.3, *p*=0.031), and degree to which symptoms were affecting or interfering with life in general in those with IBS-D (-9.0; 95% CI -17.3 to -0.8, *p*=0.032).

### Tolerability of Amitriptyline According to IBS Subtype

The total ASEC score was similar across treatment arms at 6 months for IBS-C and IBS-M or IBS-U, but higher in those with IBS-D receiving amitriptyline (Table 4). Individual treatment-emergent adverse events at 6 months, according to the ASEC, by IBS subtype are provided in Supplementary Figures 4 to 6. Excess adverse events with amitriptyline were similar across IBS subtypes, consisting mainly of those related to anticholinergic effects, including dry mouth, drowsiness, blurred vision, and urination problems. Rates of constipation were similar with amitriptyline and placebo in IBS-C (78.6% vs. 78.3%) and IBS-M or IBS-U (68.1% vs. 68.1%), but higher (>10% absolute difference) with amitriptyline in IBS-D (33.3% vs. 21.7%). Rates of diarrhoea were similar with amitriptyline and placebo in IBS-C (32.1% vs. 34.8%) and IBS-M or IBS-U (58.3% vs. 63.8%), but higher with placebo in IBS-D (71.2% vs. 85.0%).

## Discussion

We performed a series of *post hoc* analyses of the ATLANTIS trial of low-dose amitriptyline for IBS. Amitriptyline was effective overall, and across multiple endpoints, as reported previously. All patient subgroups had intervention effects in the direction of benefit, and although the magnitude of effect varied by subgroup, differences in treatment effects were not statistically significant according to most of the effect modifiers examined. There was evidence of a quantitative difference in effectiveness of amitriptyline in those ≥50 years and those in the 70% most deprived areas of England, according to IMD decile. In addition, stronger treatment effects occurred consistently in men, those with higher PHQ-12 scores, and those with IBS-D, compared with IBS-M or IBS-U. The mean differences on the IBS-SSS between amitriptyline and placebo observed in these analyses were of a magnitude comparable to the proposed minimum clinically important difference.[31, 32] In terms of patient characteristics that affected likelihood of response to amitriptyline or placebo, only age ≥50 years, compared with <50 years, predicted response to amitriptyline according to SGA of relief of IBS symptoms, and severe symptoms on the IBS-SSS, compared with mild symptoms, an increased likelihood of response to amitriptyline for a ≥30% improvement in abdominal pain. There were no significant predictors of response to placebo. Mean differences in individual IBS-SSS scores favoured amitriptyline for all components of the IBS-SSS across almost all IBS subtypes, suggesting that the drug does not worsen symptoms in any particular subtype. This was borne out by the analysis of treatment-emergent adverse events by subtype.

There was a quantitative difference in effectiveness of amitriptyline in those aged ≥50 compared with <50 years. Previous work has shown that placebo response rates are higher in younger patients with gastrointestinal disorders.[33] However, placebo response rates according to age in ATLANTIS were not markedly different, but rather response rates to active drug were. Others have shown that mean serum concentration to drug dose ratios of TCAs increase with age,[34] meaning that exposure to active drug may have been higher in older, compared with younger, patients in the trial. The depression literature suggests that response to antidepressants may be better in those of higher socioeconomic status, and it has been presumed this relates to improved adherence in this patient group.[35] Our results suggest the reverse is the case when using antidepressants at low dose for IBS. It may be that adherence was better at lower dose and that the increased attention that those with higher deprivation indices received as part of the trial influenced outcomes. The larger treatment effect seen in men seemed to be related to higher placebo response rates among women in the trial, despite similar response rates to active drug, although evidence for the influence of sex on placebo response rates is inconsistent.[33] Our finding that stronger treatment effects, and higher rates of constipation, occurred in those with IBS-D is in keeping with the known effects of TCAs, which slow gastrointestinal transit.[36, 37] Finally, higher PHQ-12 scores were associated with greater treatment effects with amitriptyline, which is in keeping with the findings of a RCT of the TCA imipramine in 138 patients with functional somatic syndromes, of whom 43 met criteria for IBS, in which improvement with imipramine was significantly greater than with placebo across all syndromes.[38]

Although we used formal interaction testing, with 10 subgroup interaction tests across three outcomes, one to two statistically significant interaction tests at the 5% level would be expected based on chance alone. The subgroup effect for age was, however, observed consistently across the primary, key secondary, and exploratory outcomes at the 10% significance level, and the direction of effect was consistent in sensitivity analysis, in which the linear, rather than the subgroup effect, of age was considered. It is well known that interaction tests are not very sensitive and the power to detect a significant treatment-by-subgroup interaction tends to be low. A non-significant interaction does not generally indicate that the treatment effect is, in fact, homogeneous across the subgroups of interest. Therefore, we also reported consistent subgroup effects observed across outcomes that did not meet statistical levels of significance. These findings should be considered as exploratory and hypothesis-generating, intended to guide future research, rather than providing definitive conclusions. We used a 6-month duration of treatment, longer than most drug trials in IBS, which usually assess efficacy over 12 weeks. We also used outcomes that are accepted widely in more pragmatic trials conducted in IBS, appropriate to the primary care setting, such as MDs in total IBS-SSS and SGA of relief symptoms of IBS. We conducted analyses on all participants, with imputation of missing data, and recruited participants irrespective of IBS subtype and demographic characteristics, allowing us to examine the impact of these on effectiveness and tolerability of drug or placebo.

Limitations include the fact that we did not utilise primary endpoints recommended by the Food and Drug administration (FDA) or European Medicines Agency (EMA) for drug trials conducted in IBS.[39, 40] Given the pragmatic nature of the trial, the requirement to answer daily symptom questions during 6 months of treatment to assess these would have been impractical and burdensome to participants. In addition, for IBS-M or IBS-U there is no consensus on endpoints. Our choice of a ≥30% improvement in abdominal pain on the IBS-SSS, however, approximates FDA and EMA-recommended endpoints. The fact that over 80% of participants had IBS-D or IBS-M means that making judgements about the effectiveness of low-dose amitriptyline in those with IBS-C is limited by the smaller participant numbers. It may be that patients with IBS-C were aware that constipation is a potential side effect of amitriptyline, although satisfaction with bowel habit did not deteriorate in those with IBS-C during the trial, and rates of constipation were not increased with active drug compared with placebo.

Nevertheless, other demographic characteristics of recruited individuals were wide ranging. Although the beneficial effect of amitriptyline varied across levels of the candidate effect modifiers, few interaction effects were statistically significant at the 5% level. This may relate to a lack of power to detect differences between subgroups. A final issue is the possibility of unblinding due to the anticholinergic side effects of amitriptyline.

Previous meta-analyses have suggested a benefit of TCAs in IBS,[11–13] but an individual patient data meta-analysis to assess patient factors that might influence response to active drug or placebo has never been conducted. In addition, most trials of TCAs have been small and have not reported on predictors of response. In the largest trial of a TCA conducted prior to ATLANTIS, which randomised 216 participants with IBS or other functional bowel disorders to either desipramine or placebo, the response to desipramine appeared to be greater in those with normal depression scores, those with moderate, compared with severe, symptoms, and those with IBS-D.[41] In our study, effectiveness according to depression scores was inconsistent across endpoints. Response to amitriptyline was greater in those with severe symptoms when the mean change in IBS-SSS or a ≥30% improvement in abdominal pain were used, but when SGA of relief was used the effect appeared greatest in those with mild symptoms. Finally, and in accordance with Drossman *et al*., greater treatment effects with amitriptyline were observed consistently in those with IBS-D across all three endpoints studied, compared with IBS-M or IBS-U. However, as discussed, individual symptom domains on the IBS-SSS improved across all IBS subtypes, relative to placebo.

We believe it is unlikely that another large placebo-controlled trial of a TCA in IBS will be conducted. Although these analyses are exploratory, the consistent effects observed according to some patient characteristics suggest subgroups of patients in whom the effectiveness of amitriptyline may be enhanced, who could either be targeted for future treatment with a TCA in clinical practice or selected for further mechanistic studies of this class of drugs. They also reveal that amitriptyline did not worsen gastrointestinal symptoms, and was well-tolerated, in those with IBS-C, IBS-M, or IBS-U. These analyses are, therefore, likely to be useful. Take home messages for clinical practice include the fact that amitriptyline’s effects appeared greatest in older individuals, those reporting multiple other somatic symptoms, men, and those with IBS-D. However, as the drug appeared of benefit for all IBS symptoms studied and well tolerated, irrespective of subtype, it would seem reasonable to offer it to patients regardless of predominant bowel habit.

## Supplementary Material

Supplement 1

Supplement 2

## Figures and Tables

**Table 1:** Treatment effect on the Total IBS-SSS Score and for SGA of Relief of IBS Symptoms at 6 Months According to Participant Baseline Characteristics.

Baseline Characteristic^‡^	Total IBS-SSS Score at 6 Months				SGA of Relief of IBS Symptoms at 6 Months			
	Mean (SD)		Adjusted Mean Difference* (Amitriptyline/ Placebo) (95% CI), *p*-value	Test for Interaction *p*-value	n (%)		Adjusted Odds Ratio^†^ (Amitriptyline/ Placebo) (95% CI), *p*-value	Test for Interaction p-value
	Amitriptyline (n=232)	Placebo (n=231)			Amitriptyline (n=232)	Placebo (n=231)		
**Age (median cut off)**								
<50 years (n=233, 50.3%)	197.1 (100.3), n=96	205.5 (112.2), n=96	-7.1 (-35.0, 20.8), *p*= 0.62		52/96 (54.2%)	46/95 (48.4%)	1.24 (0.70, 2.18), *p*=0.47	
≥50 years (n=230, 49.7%)	146.7 (109.0), n=108	195.0 (116.9), n=101	-46.5 (-74.2, -18.8), *p*=0.0010	*p*=0.048	73/108 (67.6%)	42/100 (42.0%)	2.59 (1.47, 4.55), *p*=0.0010	*p*=0.068
**Sex**								
Male (n=148, 32.0%)	161.1 (103.2), n=71	197.9 (113.1), n=61	-47.9 (-82.6, -13.2), *p*=0.0069		44/71 (62.0%)	23/60 (38.3%)	2.49 (1.24, 5.00), *p*=0.011	
Female (n=315, 68.0%)	175.3 (110.1), n=133	201.1 (115.5), n=136	-17.1 (-41.4, 7.1), *p*=0.17	*p*=0.15	81/133 (60.9%)	65/135 (48.1%)	1.53 (0.93, 2.49), *p*=0.091	*p*=0.26
**IMD decile^‡^ (median cut off)**								
≤7 (n=234, 51.0%)	166.0 (102.7), n=107	206.2 (118.7), n=93	-39.1 (-66.6, -11.6), *p*=0.0054		70/107 (65.4%)	38/91 (41.8%)	2.29 (1.30, 4.03), *p*=0.0043	
>7 (n=225, 49.0%)	172.6 (113.1), n=95	195.7 (110.7), n=103	-19.4 (-47.7, 9.0), *p*=0.18	*p*=0.33	55/95 (57.9%)	49/103 (47.6%)	1.51 (0.87, 2.61), *p*=0.15	*p*=0.29
**IBS subtype**								
IBS-C (n=77, 16.6%)	165.3 (102.5), n=36	239.0 (119.8), n=31	-50.2 (-99.1, -1.3), *p*=0.044		18/36 (50.0%)	12/31 (38.7%)	1.56 (0.58, 4.20), *p*=0.38	
IBS-D (n=181, 39.1%)	163.8 (103.7), n=81	199.0 (110.8), n=77	-38.7 (-69.7, -7.6), *p*=0.015	*p*=0.15	52/81 (64.2%)	32/76 (42.1%)	2.27 (1.21, 4.26), *p*=0.010	*p*=0.95
IBS-M or IBS-U (n=205, 44.3%)	178.6 (114.0), n=87	187.5 (113.9), n=89	-8.0 (-38.1, 22.2), *p*=0.60	*p*=0.17	55/87 (63.2%)	44/88 (50.0%)	1.50 (0.82, 2.74), *p*=0.18	*p*=0.35
**IBS severity**								
Mild (75-174) (n=63, 13.8%)	117.5 (84.2), n=32	132.6 (83.9), n=23	-1.7 (-56.5, 53.1), *p*=0.95		22/32 (68.8%)	9/23 (39.1%)	3.03 (0.99, 9.30), *p*=0.053	
Moderate (175-299) (n=201, 44.2%)	168.9 (101.3), n=91	174.0 (89.0), n=93	-14.8 (-44.9, 15.3), *p*=0.34	*p*=0.69	53/91 (58.2%)	44/92 (47.8%)	1.65 (0.91, 2.96), *p*=0.097	*p*=0.34
Severe (300-500) (n=191, 42.0%)	197.6 (116.0), n=78	257.3 (123.9), n=78	-51.6 (-84.2, -18.9), *p*=0.0020	*p*=0.13	48/78 (61.5%)	32/77 (41.6%)	1.73 (0.92, 3.24), *p*=0.087	*p*=0.39
**HADS-anxiety score**								
Normal (<8) (n=245, 52.9%)	154.2 (101.4), n=110	198.5 (119.6), n=102	-38.4 (-65.3, -11.5), *p*=0.0052		69/110 (62.7%)	47/102 (46.1%)	1.73 (1.00, 2.97), *p*=0.049	
Borderline/abnormal (≥8) (n=218, 47.1%)	189.4 (112.3), n=94	201.8 (109.3), n=95	-13.6 (-43.5, 16.4), *p*=0.37	*p*=0.23	56/94 (59.6%)	41/93 (44.1%)	1.84 (1.03, 3.26), *p*=0.038	*p*=0.88
**HADS-depression score**								
Normal (<8) (n=390, 84.2%)	168.4 (105.9), n=170	195.1 (113.2), n=169	-19.6 (-41.0, 1.8), *p* 0.073		105/170 (61.8%)	75/167 (44.9%)	1.80 (1.17, 2.77), *p*=0.0076	
Borderline/abnormal (>8) (n=73, 15.8%)	180.6 (117.7), n=34	230.4 (119.6), n=28	-65.7 (-116.0, -15.4), *p*=0.011	*p*=0.096	20/34 (58.8%)	13/28 (46.4%)	1.61 (0.59, 4.39), *p*=0.36	*p*=0.83
**PHQ-12 score (median cut off)**								
≤6 (n=256, 56.0%)	162.0 (97.0), n=115	175.5 (109.8), n=106	-10.7 (-37.9, 16.5), *p*=0.44		70/115 (60.9%)	54/106 (50.9%)	1.39 (0.82, 2.35), *p*=0.22	
>6 (n=201, 44.0%)	184.6 (119.1), n=87	230.1 (113.6), n=90	-45.8 (-75.8, -15.9), *p*=0.0027	*p*=0.092	53/87 (60.9%)	34/89 (38.2%)	2.32 (1.26, 4.28), *p*=0.0071	*p*=0.21
**WSAS score**								
Low impairment (<10) (n=210, 47.4%)	149.4 (106.6), n=104	172.6 (105.4), n=86	-19.0 (-48.4, 10.4), *p*=0.20		65/104 (62.5%)	42/85 (49.4%)	1.59 (0.88, 2.87), *p*=0.12	
Moderate impairment (10-20) (n=174, 39.3%)	188.7 (98.4), n=67	216.0 (122.0), n=78	-23.9 (-55.9, 8.2), *p*=0.14	*p*=0.83	39/67 (58.2%)	36/78 (46.2%)	1.50 (0.78, 2.87), *p*=0.22	*p*=0.90
Severe impairment (>20) (n=59, 13.3%)	223.7 (114.2), n=27	256.7 (90.8), n=21	-39.8 (-97.1, 17.4), *p*=0.17	*p*=0.52	17/27 (63.0%)	6/21 (28.6%)	3.30 (1.00, 10.85), *p*=0.050	*p*=0.28
**Time from diagnosis (median cut off)**								
≤10 years (n=253, 54.8%)	166.9 (106.2), n=108	188.7 (109.8), n=106	-24.7 (-52.3, 2.8), *p*=0.078		66/108 (61.1%)	52/106 (49.1%)	1.58 (0.92, 2.72), *p*=0.10	
>10 years (n=209, 45.2%)	174.4 (109.8), n=96	211.9 (118.7), n=90	-29.0 (-57.7, -0.4), *p*=0.047	*p*=0.83	59/96 (61.5%)	36/88 (40.9%)	2.04 (1.13, 3.69), *p*=0.019	*p*=0.53

* Mean differences estimated using linear regression adjusted for covariates (baseline IBS-SSS score, IBS subtype, sex, HADS-depression score, recruiting hub) using subgroup by treatment interaction effect and multiple imputation of missing data. Mean differences in IBS-SSS score are for amitriptyline versus placebo for each of the baseline characteristics. Negative mean differences favour amitriptyline.

† Odds ratios estimated using logistic regression adjusted for covariates (baseline IBS-SSS score, IBS subtype, sex, HADS-depression score, recruiting hub) using subgroup by treatment interaction effect and multiple imputation of missing data. Odds ratios are for amitriptyline versus placebo for each of the baseline characteristics. Odds ratios greater than 1 favour amitriptyline.

‡ In all participants, including those with missing total IBS-SSS score or SGA of relief at 6 months.

⁑ Higher deciles = less deprived.

**Table 2:** Treatment effect for a ≥30% Improvement in Abdominal Pain at 6 Months According to Participant Baseline Characteristics.

Baseline Characteristic^†^	≥30% Improvement in Abdominal Pain at 6 Months			
	n (%)		Adjusted Odds Ratio* (Amitriptyline/ Placebo) (95% CI), _*p*-value	Test for interaction *p*-value
	Amitriptyline (n=232)	Placebo (n=231)		
**Age (median cut off)**				
<50 years (n=233, 50.3%)	47/96 (49.0%)	40/96 (41.7%)	1.30 (0.74, 2.27), *p*=0.36	
≥50 years (n=230, 49.7%)	66/107 (61.7%)	44/101 (43.6%)	2.10 (1.20, 3.68), *p*=0.0093	*p*=0.23
**Sex**				
Male (n=148, 32.0%)	39/71 (54.9%)	22/61 (36.1%)	2.14 (1.05, 4.37), *p*=0.037	
Female (n=315, 68.0%)	74/132 (56.1%)	62/136 (45.6%)	1.48 (0.91, 2.40), *p*=0.12	*p*=0.41
**IMD decile^‡^ (median cut off)**				
≤7 (n=234, 51.0%)	68/106 (64.2%)	34/93 (36.6%)	2.70 (1.52, 4.77), *p*=0.0007	
>7 (n=225, 49.0%)	45/95 (47.4%)	49/103 (47.6%)	1.07 (0.62, 1.86), *p*=0.80	*p*=0.021
**IBS subtype**				
IBS-C (n=77, 16.6%)	17/36 (47.2%)	12/31 (38.7%)	1.41 (0.52, 3.80), *p*=0.49	
IBS-D (n=181, 39.1%)	48/81 (59.3%)	30/77 (39.0%)	2.28 (1.22, 4.27), *p*=0.010	*p*=0.92
IBS-M or IBS-U (n=205, 44.3%)	48/86 (55.8%)	42/89 (47.2%)	1.33 (0.73, 2.41), *p* 0.35	*p*=0.23
**IBS severity**				
Mild (75-174) (n=63, 13.8%)	12/32 (37.5%)	8/23 (34.8%)	1.08 (0.35, 3.36), *p*=0.90	
Moderate (175-299) (n=201, 44.2%)	48/91 (52.7%)	42/ 93 (45.2%)	1.39 (0.78, 2.50), *p*=0.27	*p*=0.70
Severe (300-500) (n=191, 42.0%)	52/77 (67.5%)	33/78 (42.3%)	2.56 (1.35, 4.88), *p*=0.0042	*p*=0.19
**HADS-anxiety score**				
Normal (<8) (n=245, 52.9%)	61/109 (56.0%)	46/102 (45.1%)	1.51 (0.88, 2.60), *p*=0.13	
Borderline/abnormal (≥8) (n=218, 47.1%)	52/94 (55.3%)	38/95 (40.0%)	1.82 (1.02, 3.24), *p* 0.043	*p*=0.65
**HADS-depression score**				
Normal (<8) (n=390, 84.2%)	95/169 (56.2%)	73/169 (43.2%)	1.65 (1.07, 2.54), *p*=0.024	
Borderline/abnormal (>8) (n=73, 15.8%)	18/34 (52.9%)	11/38 (39.3%)	1.67 (0.60, 4.65), *p*=0.32	*p*=0.98
**PHQ-12 score (median cut off)**				
≤6 (n=256, 56.0%)	62/115 (53.9%)	51/106 (48.1%)	1.24 (0.72, 2.12), *p*=0.44	
>6 (n=201, 44.0%)	49/86 (57.0%)	33/90 (36.7%)	2.31 (1.26, 4.22), *p*=0.0065	*p*=0.13
**WSAS score**				
Low impairment (<10) (n=210, 47.4%)	52/104 (50.0%)	39/86 (45.3%)	1.21 (0.67, 2.17), *p*=0.52	
Moderate impairment (10-20) (n=174, 39.3%)	41/67 (61.2%)	34/78 (43.6%)	1.88 (0.98, 3.59), *p*=0.057	*p*=0.32
Severe impairment (>20) (n=59, 13.3%)	15/26 (57.7%)	7/21 (33.3%)	2.50 (0.73, 8.49), *p*=0.14	*p*=0.30
**Time from diagnosis (median cut off)**				
<10 years (n=253, 54.8%)	64/107 (59.8%)	45/106 (42.5%)	1.94 (1.14, 3.31), *p*=0.014	
>10 years (n=209, 45.2%)	49/96 (51.0%)	39/90 (43.3%)	1.36 (0.76, 2.43), *p*=0.30	*p*=0.37

* Odds ratios estimated using logistic regression adjusted for covariates (baseline IBS-SSS score, IBS subtype, sex, HADS-depression score, recruiting hub) using subgroup by treatment interaction effect and multiple imputation of missing data. Odds ratios are for amitriptyline versus placebo for each of the baseline characteristics. Odds ratios greater than 1 favour amitriptyline.

† In all participants, including those with missing ≥30% improvement in abdominal pain at 6 months.

‡ Higher deciles = less deprived.

**Table 3:** Mean Difference in Individual Components of the IBS-SSS Score According to IBS Subtype.

	Mean 6-month IBS-SSS Component Score (SD)		Adjusted Mean Difference* (Amitriptyline/Placebo) (95% CI), *p*-value	Test for Interaction *p*-value
IBS-SSS Component^†^	Amitriptyline (n=232)	Placebo (n=231)		
**Abdominal pain severity**				
IBS-C	25.0 (25.9), n=36	41.0 (27.1), n=31	-10.9 (-23.3, 1.4), *p*=0.083	*p*=0.22
IBS-D	27.5 (26.9), n=81	33.0 (27.1), n=77	-7.3 (-15.3, 0.8), *p=0.077*	*p*=0.33
IBS-M or IBS-U	30.8 (27.3), n=87	32.0 (28.4), n=89	-1.7 (-9.4, 6.0), *p*=0.67	
**Abdominal pain frequency**				
IBS-C	24.7 (29.3), n=36	39.4 (29.5), n=31	-8.9 (-22.1, 4.2), *p*=0.18	*p*=0.14
IBS-D	23.2 (28.0), n=81	32.3 (30.5), n=77	-9.2 (-17.4, -1.0), *p*=0.028	*p*=0.045
IBS-M or IBS-U	29.7 (29.9), n=87	27.5 (26.9), n=89	2.5 (-5.5, 10.5), *p*=0.54	
**Abdominal distension severity**				
IBS-C	30.8 (30.3), n=36	51.3 (31.3), n=31	-15.0 (-27.6, -2.3), *p*=0.021	*p*=0.10
IBS-D	28.4 (24.4), n=81	36.2 (28.3), n=77	-7.1 (-15.4, 1.1), *p*=0.090	*p*=0.42
IBS-M or IBS-U	33.4 (29.0), n=87	37.0 (28.7), n=89	-2.6 (-10.4, 5.2), *p*=0.51	
**Satisfaction with bowel habit**				
IBS-C	50.0 (25.1), n=36	65.5 (25.4), n=31	-13.6 (-26.0, -1.3), *p*=0.031	*p*=0.17
IBS-D	48.4 (27.2), n=81	53.0 (26.6), n=77	-5.1 (-12.8, 2.6), *p*=0.20	*p*=0.75
IBS-M or IBS-U	48.6 (27.5), n=87	51.2 (26.5), n=89	-3.3 (-10.8, 4.2), *p*=0.38	
**How much affecting or interfering with life in general**				
IBS-C	34.7 (27.0), n=36	41.9 (26.8), n=31	-3.3 (-15.9, 9.2), *p*=0.60	*p*=0.80
IBS-D	36.3 (27.0), n=81	44.4 (27.8), n=77	-9.0 (-17.3, -0.8), *p*=0.032	*p*=0.18
IBS-M or IBS-U	36.1 (28.4), n=87	39.8 (28.0), n=89	-1.5 (-9.2, 6.2), *p*=0.71	

* Mean differences estimated using linear regression adjusted for covariates (baseline IBS-SSS score, IBS subtype, sex, HADS-depression score, recruiting hub) using subgroup by treatment interaction effect and multiple imputation of missing data. Mean differences in IBS-SSS score are for amitriptyline versus placebo for each IBS-SSS component according to IBS subtype. Negative mean differences favour amitriptyline.

† In all participants, including those with missing total IBS-SSS score at 6 months.

**Table 4:** Mean Difference in ASEC Scores According to IBS Subtype.

	Mean 6-month ASEC Score (SD)*	
IBS Subtype	Amitriptyline (n=166)	Placebo (n=152)
**IBS-C**	9.1 (5.7), n=28	9.4 (6.2), n=23
**IBS-D**	9.2 (6.6), n=66	7.4 (5.5), n=60
**IBS-M or IBS-U**	9.6 (5.8), n=72	9.5 (6.7), n=69

* There were 338 participants (174 amitriptyline, 164 placebo) still on treatment at 6 months, of whom 318 (166 amitriptyline, 152 placebo) completed the ASEC.

## Data Availability

All data requests should be submitted to the corresponding author, Professor Alexander C. Ford, for consideration and would be subject to review by a subgroup of the trial team, which will include the data guarantor, Professor Amanda J. Farrin. Access to anonymised data may be granted following this review. All data-sharing activities would require a data-sharing agreement.

## Study Highlights

### What is already known about this subject

- Low-dose amitriptyline, a tricyclic antidepressant (TCA) was more effective than placebo across a range of symptom measures as a second-line treatment for irritable bowel syndrome (IBS) in the ATLANTIS trial.
- Whether the beneficial effects of TCAs in IBS are affected by other factors is unclear, because most trials have been small and unable to examine these issues.
- In addition, TCAs may have deleterious effects on symptoms for some patients, such as exacerbating constipation in those with IBS with constipation or IBS with mixed bowel habits.

### What are the new findings

- In *post hoc* exploratory analyses of ATLANTIS, there was evidence of a quantitative difference in effectiveness of amitriptyline in those ≥50 years and those in the 70% most deprived areas of England, according to index of mean deprivation.
- In addition, stronger treatment effects occurred consistently in men, those with higher Patient Health Questionnaire-12 scores, and those with IBS with diarrhoea.
- Mean differences in individual IBS severity scoring system (IBS-SSS) scores favoured amitriptyline for all components of the IBS-SSS across almost all IBS subtypes.
- Excess adverse events with amitriptyline were similar across IBS subtypes, consisting mainly of those related to anticholinergic effects.
- **How might it impact on clinical practice in the foreseeable future**
  - These exploratory analyses could help identify patients in whom amitriptyline may be more effective.
  - They also highlight there are unlikely to be deleterious effects of amitriptyline in any particular IBS subtype.
  - It would, therefore, seem reasonable to offer amitriptyline to patients with IBS regardless of predominant bowel habit.
