# Supplementary material for: Predictors of response to low-dose amitriptyline for irritable bowel syndrome and efficacy and tolerability according to subtype: Post hoc analyses from the ATLANTIS trial
Source: Gut. Author manuscript; Available in PMC 2025 May 1. (PMC7617491; doi:10.1136/gutjnl-2024-334490)
Supplement: Supplement 1 [file EMS203545-supplement-Supplement_1.pdf]

SUPPLEMENTARY METHODS

Participants

Eligible participants were 18 years and over, with a general practitioner (GP) diagnosis of IBS of any subtype, meeting the Rome IV criteria for IBS.[1] All participants had ongoing symptoms at trial entry, with a score of ≥75 on the IBS Severity Scoring System (IBS-SSS),[2] despite recommended first-line treatments.[3] The latter included one or more of dietary changes and lifestyle advice, soluble fibre, antispasmodics, laxatives, or anti-diarrhoeals. Other inclusion criteria included normal haemoglobin, white cell and platelet count, and C-reactive protein within 6 months of eligibility screening and negative anti-tissue transglutaminase antibodies. Participants could not exhibit any evidence of suicidal ideation, due to the risk of amitriptyline overdose, and had to be able to complete trial questionnaires and assessments, provide written informed consent and, if female and not post-menopausal or surgically sterile, willing to use highly effective contraception. Exclusion criteria are provided in Supplementary Table 1.

Randomisation and Masking

Participants were assigned randomly (1:1) to amitriptyline or matched placebo via a web randomisation system. Allocation was minimised, ensuring treatment arms were balanced for IBS subtype, assessed via the Bristol stool form scale,[4] presence of abnormal depression scores, using a score of 8 or more on the depression subscale of the hospital anxiety and depression scale (HADS),[5] and regional hub. All participants, GPs, investigators, and statisticians were masked fully to treatment allocation prior to data lock. Trial medication was posted to participants by Leeds Teaching Hospitals NHS Trust

pharmacy. To maintain masking, unique kit codes were used and trial medication appearance, packaging, and labelling were identical for both active treatment and placebo.

Procedures

Participants received 6 months of low-dose oral amitriptyline or placebo tablets. In addition, all participants were provided with the National Institute of Health and Care Excellence-approved British Dietetic Association first-line dietary advice sheet for IBS.[6] Usual care for IBS was provided by the participant's GP, although amitriptyline, other TCAs, or drugs contraindicated with TCAs, such as monoamine oxidase inhibitors or drugs prolonging the QT interval, could not be prescribed during the trial. Participants self-titrated the dose of trial medication using standardised written information,[7] with telephone support from a research nurse at 1 and 3 weeks to assess tolerability. They were advised to start at a dose of 10mg (one tablet) at night with dose titration over 3 weeks, up to a maximum of 30mg at night (three tablets), depending on tolerability and symptom response. However, participants could modify their dose throughout the study in response to IBS symptoms and side effects, provided they did not increase beyond 30mg per day, at night. All participants completed questionnaires at baseline, 3 months, and 6 months.

Outcome Measures

Post hoc analyses were conducted on the trial's primary outcome, the effect of amitriptyline on global IBS symptoms using the IBS-SSS, the key secondary outcome, subjective global assessment (SGA) of relief of IBS symptoms, and the exploratory outcome of a ≥30% improvement in abdominal pain from baseline, at 6-month follow-up. The IBS-SSS is a validated, participant-reported, five-item questionnaire used widely in IBS trials.[2] It measures presence, severity, and frequency of abdominal pain, presence and severity of

abdominal distension, satisfaction with bowel habit, and degree to which IBS symptoms are affecting, or interfering with, the person's life. The maximum score is 500 points: <75 points indicates remission of symptoms; 75-174 points mild symptoms; 175-299 points moderate symptoms; and 300-500 points severe symptoms. A difference of ≥35 points between trial arms has been proposed as the minimum clinically important difference on the IBS-SSS.[8, 9] The SGA asks patients to report symptoms on a 5-point Likert scale, with possible responses including "worse", "unchanged", "somewhat relieved", "considerably relieved", or "completely relieved".[10] Responders on the SGA were defined as participants reporting symptoms as at least somewhat relieved. A ≥30% improvement in abdominal pain from baseline approximates one of the endpoints recommended by both the Food and Drug Administration and European Medicines Agency for trials in IBS.[11, 12] Tolerability of treatment at 6 months was assessed using the validated Antidepressant Side Effect Checklist (ASEC) for participants still on treatment.[13] The ASEC consists of 21 potential treatment-emergent adverse effects rated on a scale of 0 (absent) to 3 (severe); we summed responses to provide a total score ranging from 0 to 63, with higher scores indicating worse overall side effects.

SUPPLEMENTARY RESULTS

Effect of Baseline Characteristics on Response to Either Amitriptyline or Placebo at 6 Months

In terms of patient characteristics that affected likelihood of response to amitriptyline or placebo, older participants (age ≥50 versus <50 years) were more likely to respond to amitriptyline according to SGA of relief (OR 1.82; 95% CI 1.04 to 3.16, *p*=0.035) (Supplementary Table 2 and Supplementary Figure 3) and with some consistent evidence according to a ≥30% improvement in abdominal pain (OR 1.68; 95% CI 0.97 to 2.91, *p*=0.065) (Supplementary Table 2). Participants with severe symptoms on the IBS-SSS, compared with mild symptoms, were more likely to respond to amitriptyline in terms of a ≥30% improvement in abdominal pain (OR 3.25; 95% CI 1.36 to 7.76, *p*=0.0080). There were no predictors of response to placebo.

SUPPLEMENTARY TABLES

Supplementary Table 1. Exclusion Criteria.

Age >60 years with no GP review in the 12 months prior to screening (due to concerns about the potential for organic gastrointestinal disease).
Meeting NICE fast-track referral criteria for suspected lower gastrointestinal cancer.[14]
A known diagnosis of celiac disease or inflammatory bowel disease.
A previous diagnosis of colorectal cancer.
Involvement in another clinical trial of an investigational medicinal product.
Pregnancy, breastfeeding, or planning to become pregnant.
Current use of, or allergy or contraindications to, a tricyclic antidepressant.

Supplementary Table 2. Modifier Effect on Response Rates according to SGA of Relief of IBS Symptoms and a ≥30% Improvement in Abdominal Pain at 6 Months According to Treatment.

**Baseline Characteristic†**	**SGA of Relief of IBS Symptoms at 6 Months**		**≥30% Improvement in Abdominal Pain at 6 Months**	
	**Adjusted Odds Ratio* (95% CI), *p*-value**	**Test for Interaction *p*-value**	**Adjusted Odds Ratio* (95% CI), *p*-value**	**Test for Interaction *p*-value**
**Age (median cut off)**				
≥50 years vs. <50 years (reference)	Amitriptyline: 1.82 (1.04, 3.16), *p*=0.035<br>Placebo: 0.87 (0.49, 1.55), *p*=0.63	*p*=0.068	Amitriptyline: 1.68 (0.97, 2.91), *p*=0.065<br>Placebo: 1.04 (0.59, 1.81), *p*=0.90	*p*=0.23
**Sex**				
Male vs. female (reference)	Amitriptyline: 1.13 (0.63, 2.04), *p*=0.69<br>Placebo: 0.69 (0.37, 1.28), *p*=0.24	*p*=0.26	Amitriptyline: 1.04 (0.59, 1.86), *p*=0.89<br>Placebo: 0.72 (0.37, 1.40), *p*=0.33	*p*=0.41
**IMD decile‡ (median cut off)**				
>7 vs. ≤7 (reference)	Amitriptyline: 0.93 (0.53, 1.62), *p*=0.79<br>Placebo: 1.41 (0.80, 2.47), *p*=0.23	*p*=0.29	Amitriptyline: 0.60 (0.34, 1.03), *p*=0.066<br>Placebo: 1.50 (0.84, 2.68), *p*=0.17	*p*=0.021
**IBS subtype**				
IBS-C vs. IBS-M or IBS-U (reference)	Amitriptyline: 0.60 (0.28, 1.28), *p*=0.19<br>Placebo: 0.57 (0.24, 1.37), *p*=0.21	*p*=0.95	Amitriptyline: 0.71 (0.32, 1.55), *p*=0.39<br>Placebo: 0.67 (0.29, 1.52), *p*=0.34	*p*=0.92

IBS-D vs. IBS-M or IBS-U (reference)	Amitriptyline: 1.11 (0.60, 2.04), *p*=0.74<br>Placebo: 0.73 (0.40, 1.35), *p*=0.32	*p*=0.35	Amitriptyline: 1.19 (0.63, 2.23), *p*=0.59<br>Placebo: 0.69 (0.37, 1.30), *p*=0.25	*p*=0.23
**IBS severity**				
Moderate vs. mild (reference)	Amitriptyline: 0.75 (0.33, 1.72), *p*=0.50<br>Placebo: 1.39 (0.54, 3.56), *p*=0.50	*p*=0.34	Amitriptyline: 1.97 (0.85, 4.54), *p*=0.11<br>Placebo: 1.52 (0.59, 3.94), *p*=0.38	*p*=0.70
Severe vs. mild (reference)	Amitriptyline: 0.69 (0.30, 1.60), *p*=0.39<br>Placebo: 1.21 (0.46, 3.20), *p*=0.70	*p*=0.39	Amitriptyline: 3.25 (1.36, 7.76), *p*=0.0080<br>Placebo: 1.37 (0.53, 3.53), *p*=0.52	*p*=0.19
**HADS-anxiety score**				
Borderline/abnormal vs. normal (reference)	Amitriptyline: 1.01 (0.55, 1.85), *p*=0.97<br>Placebo: 0.95 (0.53, 1.69), *p*=0.86	*p*=0.88	Amitriptyline: 1.00 (0.54, 1.85), *p*=1.00<br>Placebo: 0.83 (0.46, 1.50), *p*=0.54	*p*=0.65
**HADS-depression score**				
Borderline/abnormal vs. normal (reference)	Amitriptyline: 0.98 (0.47, 2.05), *p*=0.96<br>Placebo: 1.10 (0.49, 2.47), *p*=0.81	*p*=0.83	Amitriptyline: 0.93 (0.45, 1.95), *p*=0.85<br>Placebo: 0.92 (0.39, 2.17), *p*=0.85	*p*=0.98
**PHQ-12 score (median cut off)**				
>6 vs. ≤6 (reference)	Amitriptyline: 1.03 (0.57, 1.85), *p*=0.93<br>Placebo: 0.62 (0.34, 1.12), *p*=0.11	*p*=0.21	Amitriptyline: 1.17 (0.64, 2.12), *p*=0.61<br>Placebo: 0.62 (0.34, 1.13), *p*=0.12	*p*=0.13
**WSAS score**				
Moderate vs. low (reference)	Amitriptyline: 0.74 (0.40, 1.36), *p*=0.33		Amitriptyline: 1.39 (0.76, 2.57), *p*=0.29	

	Placebo: 0.78 (0.41, 1.48), *p*=0.45	*p*=0.90	Placebo: 0.90 (0.48, 1.68), *p*=0.73	*p*=0.32
Severe vs. low (reference)	Amitriptyline: 0.98 (0.40, 2.42), *p*=0.97		Amitriptyline: 1.33 (0.54, 3.30), *p*=0.53	
	Placebo: 0.47 (0.17, 1.33), *p*=0.16	*p*=0.28	Placebo: 0.65 (0.23, 1.81), *p*=0.40	*p*=0.30
**Time from diagnosis (median cut off)**				
>10 years vs. ≤10 years (reference)	Amitriptyline: 1.04 (0.59, 1.81), *p*=0.90		Amitriptyline: 0.69 (0.39, 1.20), *p*=0.19	
	Placebo: 0.80 (0.45, 1.43), *p*=0.46	*p*=0.53	Placebo: 0.98 (0.55, 1.76), *p*=0.95	*p*=0.37

*Odds ratios estimated using logistic regression adjusted for covariates (baseline IBS-SSS score, IBS subtype, sex, HADS-depression score, recruiting hub) using subgroup by treatment interaction effect and multiple imputation of missing data. Odds ratios are for the comparator versus the reference category for each baseline characteristic for either amitriptyline or placebo. Odds ratios greater than 1 favour the comparator.

†In all participants, including those with missing SGA of relief or ≥30% improvement in abdominal pain at 6 months.

‡Higher deciles = less deprived.

Supplemental material BMJ Publishing Group Limited (BMJ) disclaims all liability and responsibility arising from any reliance placed on this supplemental material which has been supplied by the author(s) *Gut*

SUPPLEMENTARY FIGURES

Supplementary Figure 1. Forest Plot of Treatment Effects on IBS-SSS score at 6 Months According to Participant Baseline Characteristics on the Continuous Scale*.

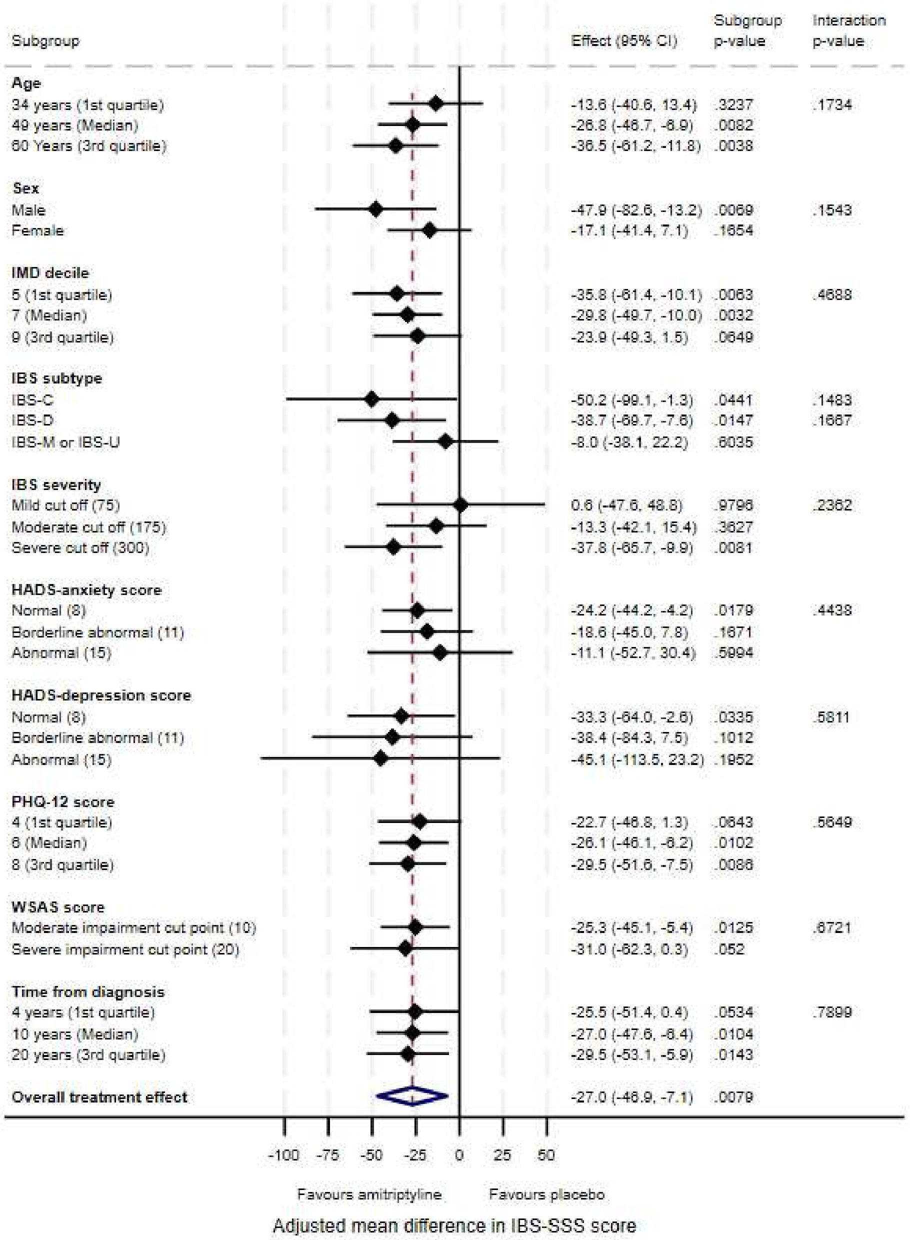

*Sensitivity analysis with age, IMD decile, IBS severity, HADS, PHQ-12, and WSAS scores and time from diagnosis fitted as continuous modifiers.

Supplementary Figure 2. Forest Plot of Treatment Effects on SGA of Relief at 6 Months According to Participant Baseline Characteristics on the Continuous Scale*.

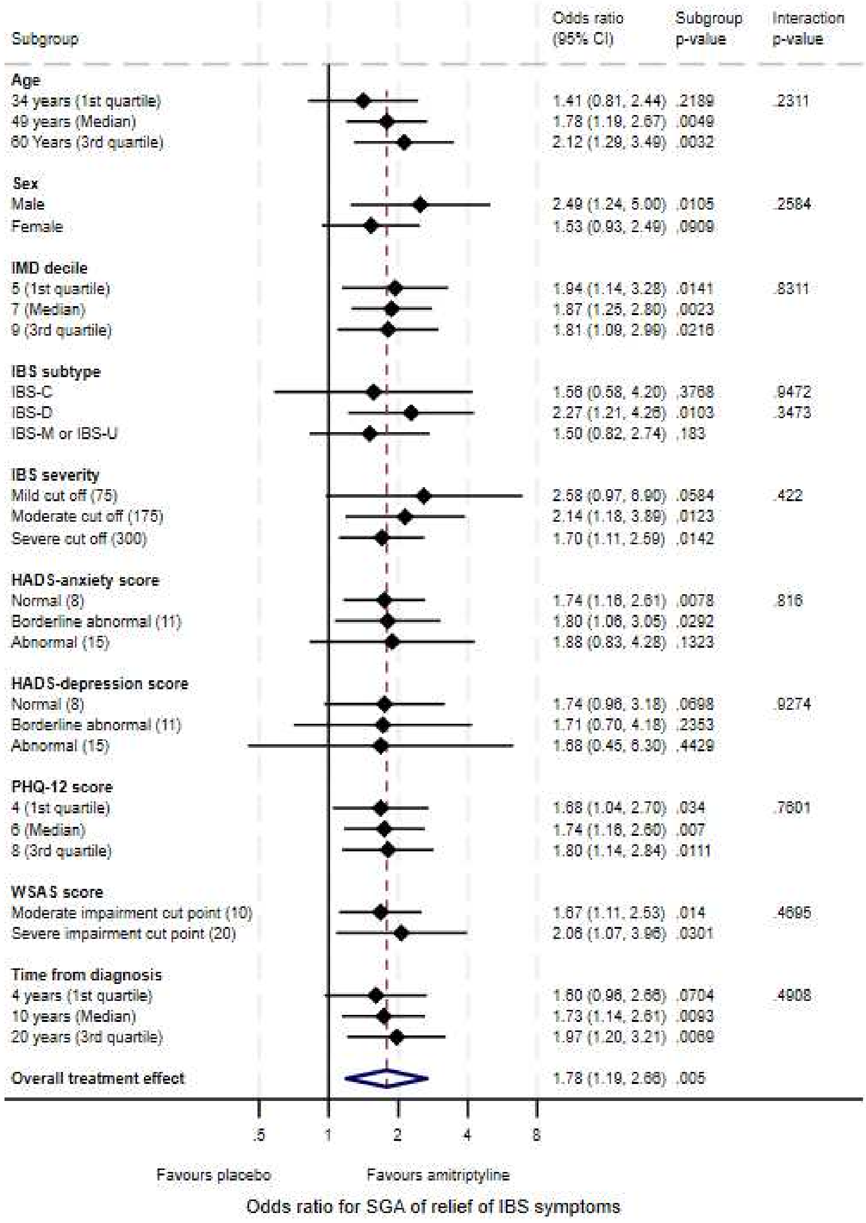

*Sensitivity analysis with age, IMD decile, IBS severity, HADS, PHQ-12, and WSAS scores and time from diagnosis fitted as continuous moderators.

Supplemental material
BMJ Publishing Group Limited (BMJ) disclaims all liability and responsibility arising from any reliance placed on this supplemental material which has been supplied by the author(s)

Supplementary Figure 3. Forest Plot of Subgroup Effects on SGA of Relief at 6 Months According to Trial Arm.

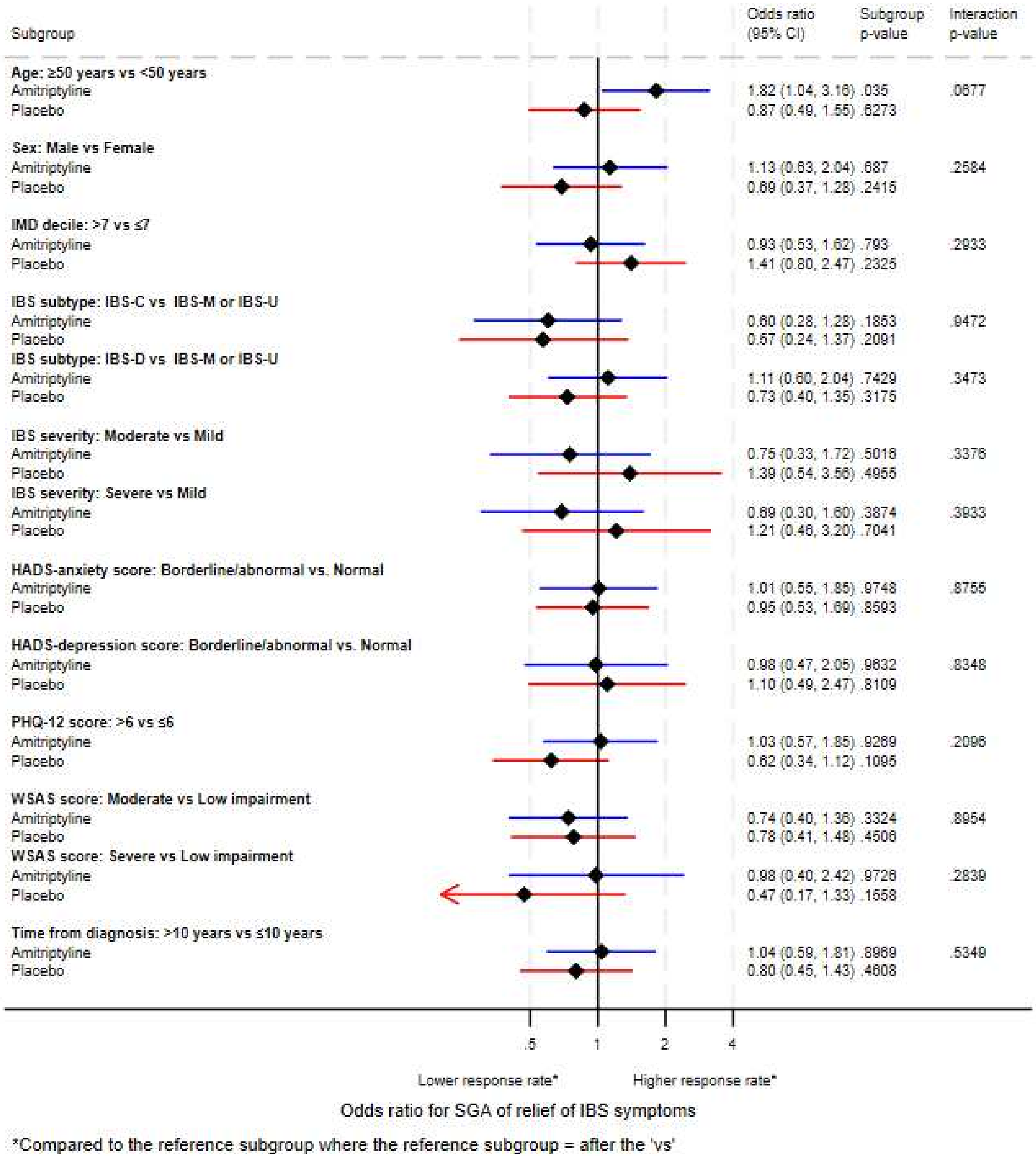

Supplementary Figure 4. Treatment-emergent Adverse Events at 6 Months in the Safety Analysis Set for Participants with IBS-C on Treatment.

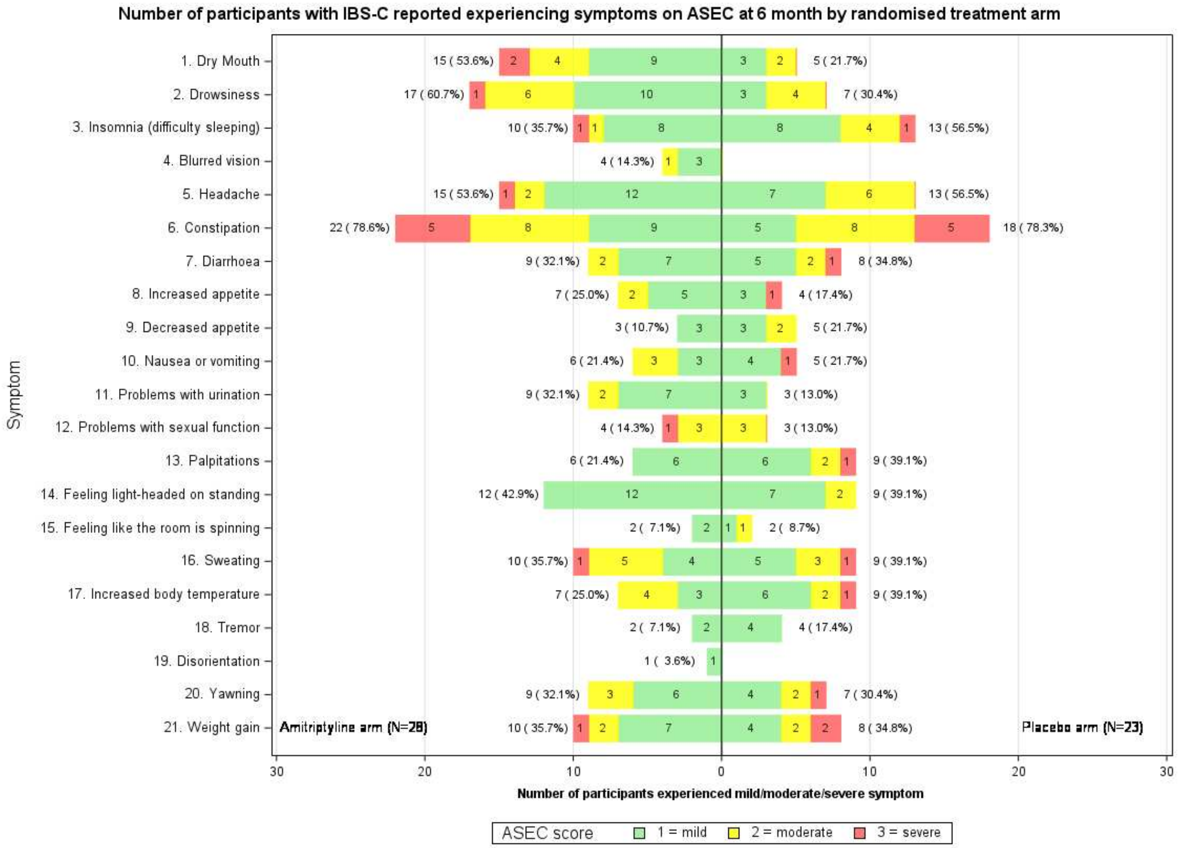

N is number of participants who were on treatment and completed the ASEC questionnaire in each arm and percentages calculated out of N

Supplementary Figure 5. Treatment-emergent Adverse Events at 6 Months in the Safety Analysis Set for Participants with IBS-D on Treatment.

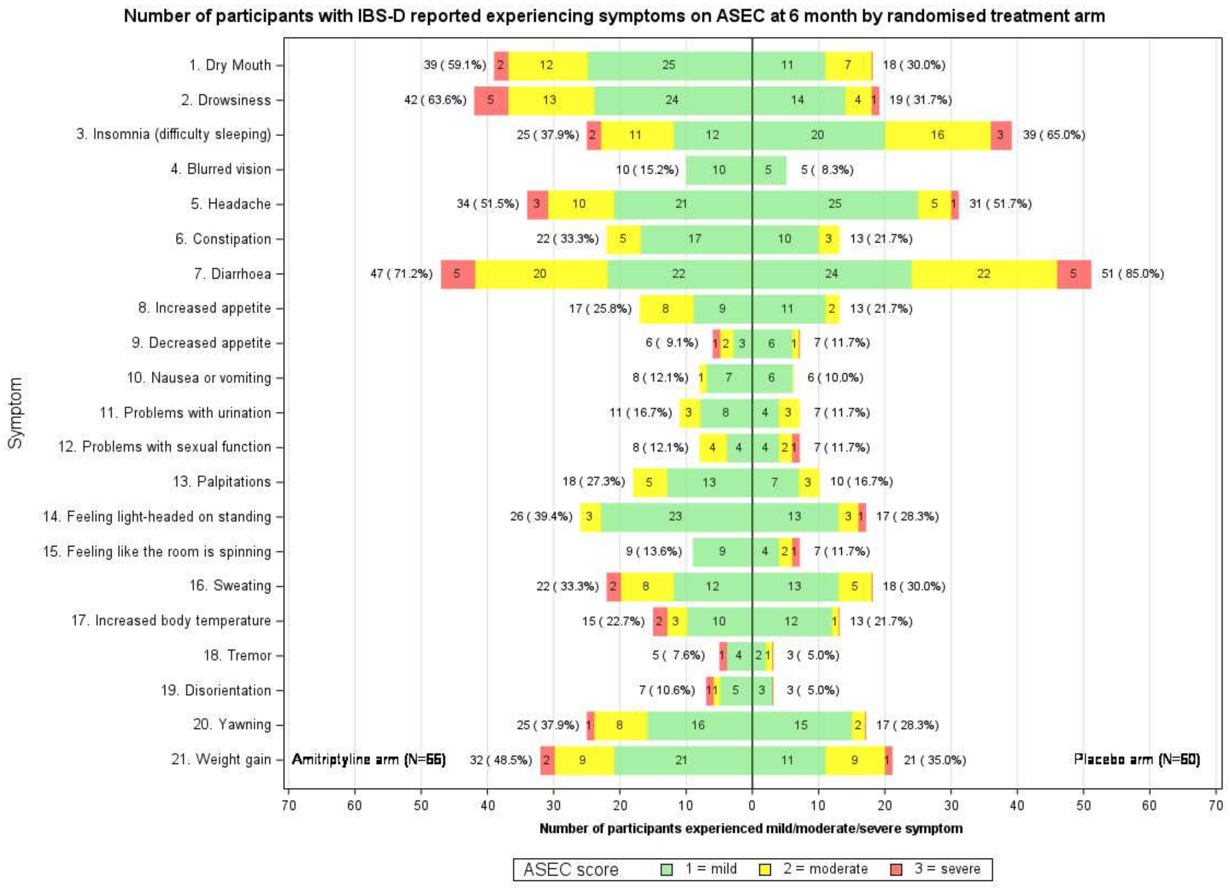

N is number of participants who were on treatment and completed the ASEC questionnaire in each arm and percentages calculated out of N

Supplementary Figure 6. Treatment-emergent Adverse Events at 6 Months in the Safety Analysis Set for Participants with IBS-M or IBS-U on Treatment.

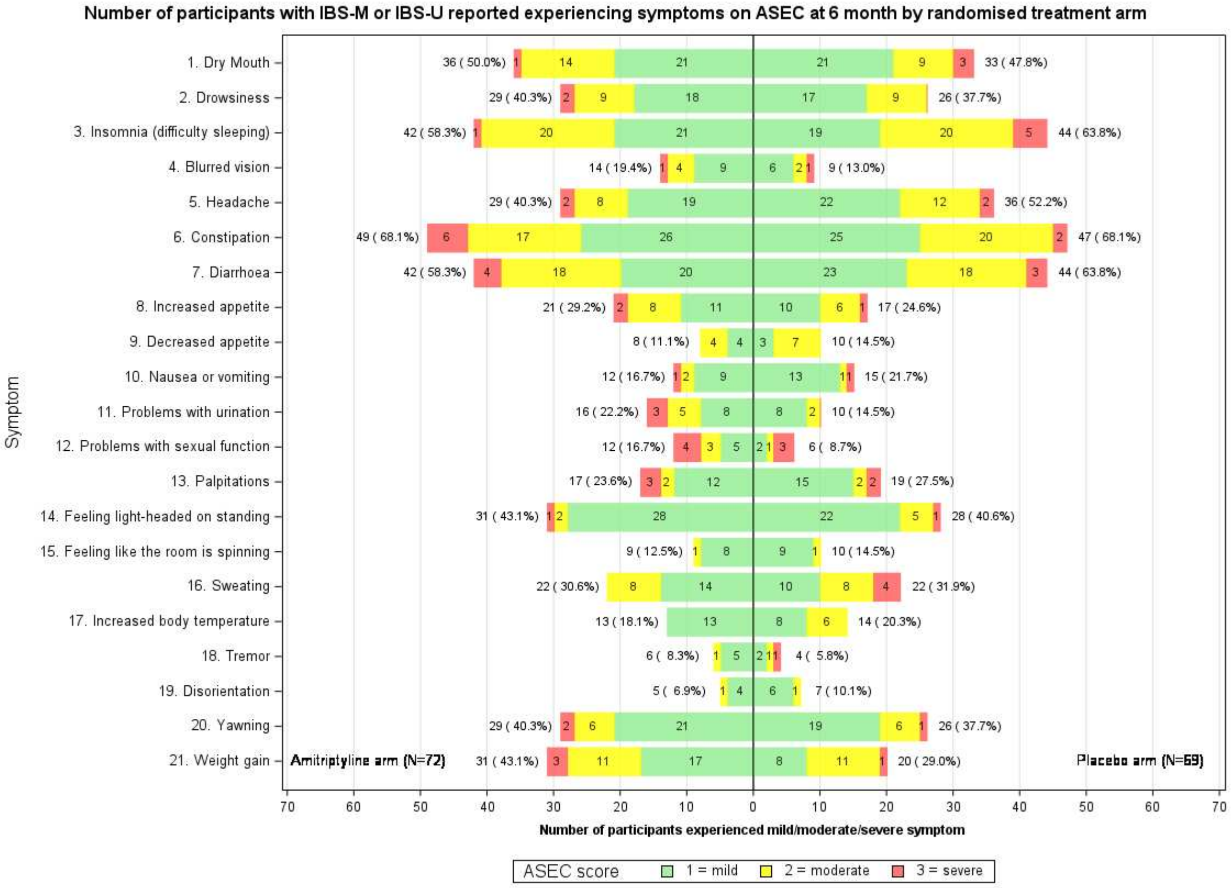

N is number of participants who were on treatment and completed the ASEC questionnaire in each arm and percentages calculated out of N

REFERENCES

1 Mearin F, Lacy BE, Chang L, Chey WD, Lembo AJ, Simren M*, et al.* Bowel disorders. Gastroenterology 2016;**150**:1393-407.

2 Francis CY, Morris J, Whorwell PJ. The irritable bowel severity scoring system: A simple method of monitoring irritable bowel syndrome and its progress. Aliment Pharmacol Ther 1997;**11**:395-402.

3 Hookway C, Buckner S, Crosland P, Longson D. Irritable bowel syndrome in adults in primary care: Summary of updated NICE guidance. BMJ 2015;**350**:h701.

4 Heaton KW, Ghosh S, Braddon FE. How bad are the symptoms and bowel dysfunction of patients with the irritable bowel syndrome? A prospective, controlled study with emphasis on stool form. Gut 1991;**32**:73-9.

5 Zigmond AS, Snaith RP. The hospital anxiety and depression scale. Acta Psychiatr Scand 1983;**67**:361-70.

6 British Dietetic Association. Irritable bowel syndrome and diet, https://www.bda.uk.com/resource/irritable-bowel-syndrome-diet.html.

7 Amitriptyline for Irritable Bowel Syndrome: Dose Adjustment Information. https://ctru.leeds.ac.uk/download/6167.

8 Everitt H, Moss-Morris R, Sibelli A, Tapp L, Coleman N, Yardley L*, et al.* Management of irritable bowel syndrome in primary care: The results of an exploratory

randomised controlled trial of mebeverine, methylcellulose, placebo and a self-management website. BMC Gastroenterol 2013;**13**:68.

9 Everitt HA, Landau S, O'Reilly G, Sibelli A, Hughes S, Windgassen S*, et al.* Assessing telephone-delivered cognitive-behavioural therapy (CBT) and web-delivered CBT versus treatment as usual in irritable bowel syndrome (ACTIB): A multicentre randomised trial. Gut 2019;**68**:1613-23.

10 Muller-Lissner S, Koch G, Talley NJ, Drossman D, Rueegg P, Dunger-Baldauf C*, et al.* Subject's Global Assessment of Relief: An appropriate method to assess the impact of treatment on irritable bowel syndrome-related symptoms in clinical trials. J Clin Epidemiol 2003;**56**:310-6.

11 Food and Drug Administration. Guidance for industry: Irritable bowel syndrome - clinical evaluation of drugs for treatment. https://www.fda.gov/media/78622/download. 2012.

12 European Medicines Agency. Guideline on the evaluation of medicinal products for the treatment of irritable bowel syndrome. https://www.ema.europa.eu/en/documents/scientific-guideline/guideline-evaluation-medicinal-products-treatment-irritable-bowel-syndrome-revision-1_en.pdf. 2014.

13 Uher R, Farmer A, Henigsberg N, Rietschel M, Mors O, Maier W*, et al.* Adverse reactions to antidepressants. Br J Psychiatry 2009;**195**:202-10.

14 Suspected cancer: recognition and referral. https://www.nice.org.uk/guidance/NG12/chapter/1-Recommendations-organised-by-site-of-cancer#lower-gastrointestinal-tract-cancers. 2015.
