# Supplementary material for: Predictors of response to low-dose amitriptyline for irritable bowel syndrome and efficacy and tolerability according to subtype: Post hoc analyses from the ATLANTIS trial
Source: Gut. Author manuscript; Available in PMC 2025 May 1. (PMC7617491; doi:10.1136/gutjnl-2024-334490)
Supplement: Supplement 2 [file EMS203545-supplement-Supplement_2.pdf]

BMJ Publishing Group Limited (BMJ) disclaims all liability and responsibility arising from any reliance placed on this supplemental material which has been supplied by the author(s)

PREDICTORS OF RESPONSE TO LOW-DOSE AMITRIPTYLINE FOR IRRITABLE BOWEL SYNDROME AND EFFICACY ACCORDING TO SUBTYPE: *POST HOC* ANALYSES FROM THE ATLANTIS TRIAL

463 patients with Rome IV IBS

232 received low-dose amitriptyline 10mg o.d. to 30mg o.d.

231 received placebo 10mg o.d. to 30mg o.d.

***Post hoc* effect modifier analysis and analysis of effect on individual symptoms of IBS and tolerability**

Predictors of response:

Increasing age (≥50 years)
Higher deprivation levels

Stronger treatment effects seen in:

Men

Higher PHQ-12 scores

IBS with diarrhoea

Improvement in individual symptoms:

Abdominal pain

Abdominal distension

Satisfaction with bowel habit

Interference with life

Higher side effect scores in IBS with diarrhoea

Wright-Hughes, et al. *Gut* 2025. DOI: 10.1136/gut-2025-334490

gut.bmj.com

@Gut_BMJ

Gut
